# Shakespeare as a Physician

**Published:** 1884-05

**Authors:** 


**Shakespeare as a Physician.** Comprising every word which in any way relates to medicine, surgery or obstetrics found in the complete works of that writer, with criticisms and comparisons of the same with the medical thoughts of to-day. By J. P. CHESNEY, M. D., Prof. of Gynæcology in the Northwestern Medical College, St. Joseph, Mo., etc. J. H. Chambers & Co., Chicago, St. Louis, and Atlanta, Ga. 1884.

The conception of presenting Shakespeare's medical knowledge in a complete and connected form is doubtless original with the author. The work, as accomplished, must have required a deal of patient study and perseverance upon the part of its writer, but he has succeeded in producing so interesting and readable a book that every physician, or, at least, every one at all familiar with the great author's works, should possess a copy.

